# Prediction of Heavy Metal Removal by Different Liner Materials from Landfill Leachate: Modeling of Experimental Results Using Artificial Intelligence Technique

**DOI:** 10.1155/2013/240158

**Published:** 2013-06-10

**Authors:** Nurdan Gamze Turan, Emine Beril Gümüşel, Okan Ozgonenel

**Affiliations:** ^1^Department of Environmental Engineering, Engineering Faculty, Ondokuz Mays University, Kurupelit, 55139 Samsun, Turkey; ^2^Department of Electric and Electronic Engineering, Engineering Faculty, Ondokuz Mays University, Kurupelit, 55139 Samsun, Turkey

## Abstract

An intensive study has been made to see the performance of the different liner materials with bentonite on the removal efficiency of Cu(II) and Zn(II) from industrial leachate. An artificial neural network (ANN) was used to display the significant levels of the analyzed liner materials on the removal efficiency. The statistical analysis proves that the effect of natural zeolite was significant by a cubic spline model with a 99.93% removal efficiency. Optimization of liner materials was achieved by minimizing bentonite mixtures, which were costly, and maximizing Cu(II) and Zn(II) removal efficiency. The removal efficiencies were calculated as 45.07% and 48.19% for Cu(II) and Zn(II), respectively, when only bentonite was used as liner material. However, 60% of natural zeolite with 40% of bentonite combination was found to be the best for Cu(II) removal (95%), and 80% of vermiculite and pumice with 20% of bentonite combination was found to be the best for Zn(II) removal (61.24% and 65.09%). Similarly, 60% of natural zeolite with 40% of bentonite combination was found to be the best for Zn(II) removal (89.19%), and 80% of vermiculite and pumice with 20% of bentonite combination was found to be the best for Zn(II) removal (82.76% and 74.89%).

## 1. Introduction

Industrial wastes are generated in large amounts in several industries. Because of their toxicity and nonbiodegradable nature, heavy metals are of special significance [1, 2]. Industrial waste containing heavy metals is being released into the nonengineered open dumps causing detrimental effects not only on humans but also upon environment; therefore it has become imperative to develop methods for treating such wastes [3].

The sanitary landfill method for the ultimate disposal of industrial waste continues to be widely accepted and used due to its economic advantages [4, 5]. Leachate is defined as the aqueous effluent generated as a consequence of rainwater percolation through wastes, biochemical processes in waste's cells, and the inherent water content of wastes themselves [6]. When water percolates through solid wastes, both biological materials and chemical constituents are leached into solution [7, 8]. The major concern with the movement of leachate into the subsurface aquifer is the fate of the constituents found in waste [9].

In landfill, technical and geological barrier systems are employed to minimize uncontrolled emissions from the waste into the environment. The barrier systems contain a liner material, which should have a low hydraulic conductivity and the ability to attenuate pollutants migrating through the barrier [10]. Liner materials must be developed or improved with respect to ecological and economical requirements. Moreover, these materials to prevent or control shrinkage and/or desiccation cracking need to be further investigated (the choice of suitable liner material, modifications affecting leachate quality, etc.).

Bentonite, which is typically clay, is widely used for liner material in the barrier system. It has local availability and a low hydraulic conductivity. However, leakage can result from shrinkage cracking if only bentonite is used [11]. For this reason, a suitable sand-bentonite mixture to determine the minimum percentage of bentonite necessary to fulfil the given requirements is the main task [12]. Previous studies showed that quantities higher than 15% of bentonite as an amendment in a mixture do not lead to a significant decrease in hydraulic conductivity, while strength properties and mechanical behaviour of the mixture may be adversely affected by the clay [13, 14].

The artificial neural network (ANN) is a system of data processing based on the structure of a biological neural system. The prediction with ANN is made by learning of the experimentally generated data or using validated models [15]. Because of their reliable, robust, and salient characteristics in capturing the nonlinear relationships existing between variables (multiinput/output) in complex systems, numerous applications of ANN have been successfully conducted to solve environmental problems [16–18]. 

In the literature, there are few studies relating to operation problems for landfilling processes based on ANNs. In the present work, heavy metal removal during landfilling of industrial waste is investigated. The effects of various liner materials, such as bentonite, natural zeolite, expanded vermiculite, and pumice on the removal of Cu(II) and Zn(II) are examined. On the basis of batch adsorption experiments, a three-layer ANN model to predict heavy metal removal efficiency of composite used as a liner material is applied in this work. Removal of heavy metal from landfilling process is optimized to determine the optimal network structure. Finally, outputs obtained from the models are compared with the experimental data, and advantages and the further developments are also discussed.

## 2. Material and Methods

### 2.1. Materials

The three natural materials and the commercially available bentonite were investigated as a liner material in this study. Among them, natural zeolitee was obtained from the Rota Mining Industry (Gördes, Manisa, Turkey); expandable vermiculite was obtained from the Fitar Agricultural Industry (Antalya, Turkey); pumice was obtained from the Soylu Mining Industry (Nevşehir, Turkey); illite was obtained from Sud Chemie Mining Industry and Trade Co. Ltd. (Ordu, Turkey); kaolinite was obtained from the Kale Mining Industry and Trade Co. Ltd. (Çanakkale, Turkey); and bentonite was obtained from the Bensan Activated Bentonite Company (Enez, Edirne, Turkey). The chemical composition of the materials is presented in Table 1. Samples were crushed and then milled resulting in small particles with a size of about 0.5 mm.

### 2.2. Experimental Procedure

The industrial waste obtained from an electroplating industry in Samsun (Turkey) was used in the experiments. Ten simulated landfill systems were used for the removal of heavy metal from leachate. The systems were composed with a capacity of 25 L (20 cm × 25 cm × 50 cm).

Natural material-bentonite mixtures were prepared to evaluate how much these clays reduce the removal of heavy metal as liner materials. The amounts of natural materials used in the mixtures were 25%, 50%, and 75% of mixtures as volumetric. The mixture of natural liner materials is placed at the base of the simulated landfill systems. Total volume of liner material was 5 L for all systems. The system containing 100% of bentonite as a liner material was compared to other systems. A 20 L of industrial waste was deposited on the liner materials. 

Leachate was collected from a drainage channel at the bottom of the system by adding distilled water. For each week, a total volume of 1 L influent was passed through the industrial waste sample. 500 mL of effluent was collected and acidified with concentrated nitric acid. The tests were conducted for 15 weeks. For each sample, the effluent was then analyzed for the heavy metal ions Cu^2+^ and Zn^2+^ using an atomic adsorption spectrophotometer (UNICAM 929 Model). Duplicate samples were prepared for all tests. 

### 2.3. Artificial Neural Network Application

An effective way to modeling batch adsorption system for Cu(II) and Zn(II) removal is achieved by the use of artificial neural network (ANN). Nowadays, considerable achievements in artificial intelligence techniques can be used to model and predict the responses in complex systems. These techniques can enhance the predicting ability of the model such as adsorption systems if the mathematical or statistical methods fail to formulate with the desired accuracy. 

A lot of scientists present ANN techniques for modeling batch experimental systems. Generally, feed-forward back propagation (FFBP) ANNs were successfully used in adsorption studies [19–24]. Details about ANNs can be found in the related literature. All these techniques use more or less the same network architecture. The optimum network type is found by trial and error, and training procedures for these suggested ANNs need long computer runs. FFBP consists of one input layer, one or several hidden layers, and one output layer. Back propagation (BP) learning algorithm is usually used for learning procedure. The mathematical background of BP algorithm can be found in [25, 26].

**Figure 1:**
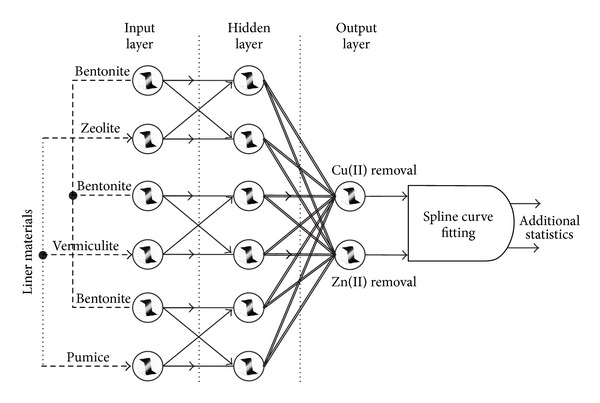
Proposed ANN structure for modeling adsorption system.

In this paper a simple ANN topology with 2 neurons in input layer, 2 neurons in hidden layer, and 2 neurons in output layer is needed to model the system. There are a number of common activation functions in use with ANNs. The most common choice of activation functions for multilayered perceptron (MLP) is used as hyperbolic tangent function (Figure 1). 

As seen in Figure 1 three liner materials were tested to see the efficiency of the proposed batch experimental system to maximize the Cu(II) and Zn(II) removal. Training process consists of four steps: (a) assemble the training data, (b) decide the network type, (c) train the network, and (d) calculate the output for test data. Unlike experimental design, the proposed ANN uses only 2 inputs and responses of 2, that is, Cu(II) and Zn(II) removal. Therefore, it is easy to implement and cost-effective. 

The input and target data were selected from Table 2. The percentage of each liner material, that is, natural zeolite, expanded vermiculite, and pumice, and removal efficiencies were used for training procedures. 

Table 2 actually was used for training the ANNs according to the network parameters given in Table 3.

**Figure 2:**
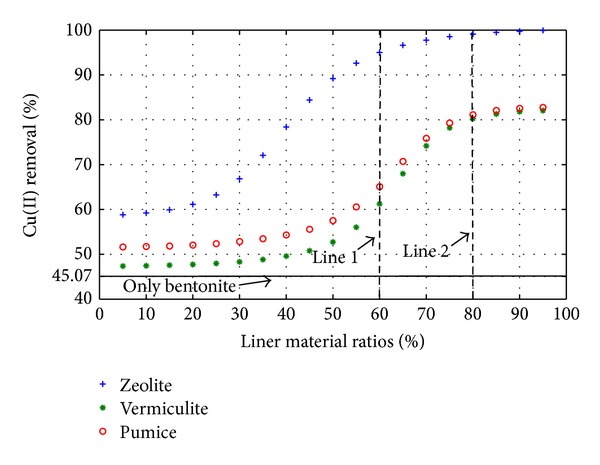
Prediction of Cu(II) removal with different liner materials.

**Figure 3:**
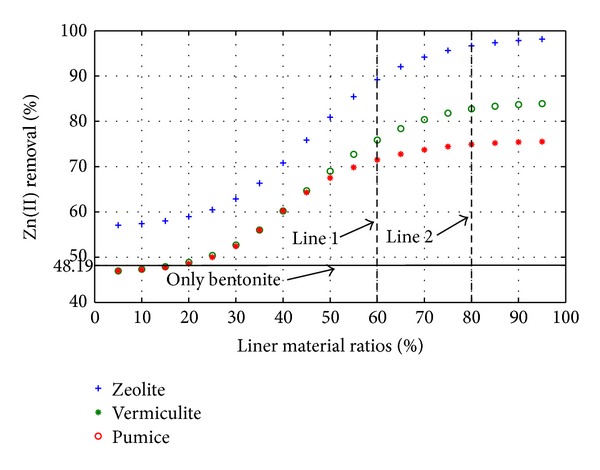
Prediction of Zn(II) removal with different liner materials.

For testing the ANNs (Figure 1) the ratio of each liner with respect to bentonite was changed from 5% to 95% and the outputs of ANNs were predicted. This testing procedure yielded to 19 trials. Three of the total trials such as 25%, 50%, and 75% of the liner materials and 75%, 50%, and 25% of bentonite were already used in testing process and these three trials were then used to check the consistency of the ANNs. Figures 2 and 3 demonstrate the performance of the suggested ANNs topology for Cu(II) and Zn(II) removal, respectively. 

In Figures 2 and 3 line 1 demonstrates the optimal point for natural zeolite material while line shows the optimal points for the materials of expanded vermiculite and pumice. Consequently, 60% of natural zeolite with 40% of bentonite combination was found to be best for Cu(II) removal (95%) and 80% of expanded vermiculite and pumice with 20% of bentonite combination was found to be the best for Zn(II) removal (61.24% and 65.09%). Similarly, 60% of natural zeolite with 40% of bentonite combination was found to be the best for Zn(II) removal (89.19%), and 80% of expanded vermiculite and pumice with 20% of bentonite combination was found to be the best for Zn(II) removal (82.76% and 74.89%). A minimum liner material and bentonite combination can be selected as 20% according to Figures 2 and 3 since there is no significant change in the removal efficiency up to that point.

The intermediate values of the outputs of ANNs can also be tested by cubic spline interpolation technique. Equation (1) gives the mathematical explanation of the interpolation technique:

(1) $$\begin{array}{c} S\left(z,v,p\right)=\{\begin{array}{c} ax^{3}+bx^{2}+cx+d,\\ ex^{3}+fx^{2}+gx+h\text{.} \end{array} \end{array}$$

In (1) *z*, *v*, and *p* stand for natural zeolite, expanded vermiculite, and pumice, respectively, and *x* represents the interval areas, that is, from 5% to 95%.  Table 4 gives the descriptive statistics of the interpolated outputs of ANNs.

The removal efficiencies were calculated as 45.07% and 48.19% for Cu(II) and Zn(II), respectively (Table 1), when only bentonite was used as liner material. However, the use of other liner materials in specific ratios had significant effect on removal efficiencies (Table 3). 

## 3. Conclusion

The idea of the study was to examine the feasibility of using different liner materials to remove Cu(II) and Zn(II) from industrial leachate. The following outcomes can be derived from this ongoing research work.

- The traditional use of bentonite as a liner material has low removal efficiency comparing to combinations of natural zeolite + bentonite, expanded vermiculite + bentonite, and pumice + bentonite mixtures.
- The suggested ANN topology was found effective to model the experimental design.
- Applying cubic spline curve fitting of the removal efficiencies enables the provision of additional descriptive statistics.
- Among the different combinations of liner materials 60% of natural zeolite + 40% of bentonite was found the optimum with high removal efficiencies of 95% for Cu(II) and 89.19% for Zn(II).

## Figures and Tables

**Table 1:** Chemical compositions of the liner materials.

Components	B	NZ	EV	P
Na_2_O	1.80	0.40	0.05	3.65
MgO	4.00	1.40	17.75	0.03
Al_2_O_3_	17.00	11.80	18.45	12.27
SiO_2_	61.00	71.00	41.29	73.44
CaO	2.50	3.40	0.25	0.96
TiO_2_	—	0.10	1.21	0.10
K_2_O	0.50	2.40	7.21	4.37
Fe_2_O_3_	3.00	1.70	6.51	1.2
MnO	—	—	0.04	0.06
SO_3_	—	0.12	—	0.08
LOI	3.51	6.87	5.02	3.72
CEC (meq/100)	31.8	166.3	52.9	34.6

B: bentonite, NZ: natural zeolite, EV: expanded vermiculite, P: pumice.

**Table 2:** The whole experimental system.

Liner materials %	Cu(II) removal (%)	Zn(II) removal (%)
Bentonite	45.07	48.19
25% natural zeolite + 75% bentonite	63.27	60.47
50% natural zeolite + 50% bentonite	89.20	80.90
75% natural zeolite + 25% bentonite	98.34	95.29
25% vermiculite + 75% bentonite	47.97	50.31
50% vermiculite + 50% bentonite	52.73	68.98
75% vermiculite + 25% bentonite	77.41	81.49
25% pumice + 75% bentonite	52.38	50.00
50% pumice + 50% bentonite	57.50	67.50
75% pumice + 25% bentonite	78.62	74.24

**Table 3:** Training parameters for all ANNs.

Max. iteration	20000
Learn rate start control iteration	1.000
Learn rate	0.075
Min. learn rate	0.001
Max. learn rate	0.075
Momentum	0.800
Tolerance	0.000
RMS error	0.000

**Table 4:** Basic statistics for interpolated ANN outputs.

Descriptive statistics	Cu(II)_natural zeolite_	Cu(II)_vermiculite_	Cu(II)_pumice_	Zn(II)_natural zeolite_	Zn(II)_vermiculite_	Zn(II)_pumice_
Minimum	58.83	47.37	51.63	57.06	46.57	46.57
Maximum	99.93	82.05	82.77	98.15	83.89	75.52
Mean	82.74	60.60	63.86	78.69	66.68	63.38
Median	89.20	52.73	57.50	80.90	68.98	67.50
Mod	58.83	47.37	51.63	57.06	46.97	46.97
Standard value	16.62	14.35	12.74	16.38	14.64	11.46
Range	41.10	34.68	31.13	41.09	36.93	28.55
